# Clinically Relevant Molecular Biomarkers for Use in Human Knee Osteoarthritis: A Systematic Review

**DOI:** 10.1177/1947603520941239

**Published:** 2020-07-17

**Authors:** James G. Convill, Gwenllian F. Tawy, Anthony J. Freemont, Leela C. Biant

**Affiliations:** 1 Faculty of Biology, Medicine and Health, University of Manchester, Manchester, UK

**Keywords:** biomarkers, molecular biomarkers, osteoarthritis, knee

## Abstract

**Objective:**

Biomarkers in osteoarthritis (OA) could serve as objective clinical indicators for various disease parameters, and act as surrogate endpoints in clinical trials for disease-modifying drugs. The aim of this systematic review was to produce a comprehensive list of candidate molecular biomarkers for knee OA after the 2013 ESCEO review and discern whether any have been studied in sufficient detail for use in clinical settings.

**Design:**

MEDLINE and Embase databases were searched between August 2013 and May 2018 using the keywords “knee osteoarthritis,” “osteoarthritis,” and “biomarker.” Studies were screened by title, abstract, and full text. Human studies on knee OA that were published in the English language were included. Excluded were studies on genetic/imaging/cellular markers, studies on participants with secondary OA, and publications that were review/abstract-only. Study quality and bias were assessed. Statistically significant data regarding the relationship between a biomarker and a disease parameter were extracted.

**Results:**

A total of 80 studies were included in the final review and 89 statistically significant individual molecular biomarkers were identified. C-telopeptide of type II collagen (CTXII) was shown to predict progression of knee OA in urine and serum in multiple studies. Synovial fluid vascular endothelial growth factor concentration was reported by 2 studies to be predictive of knee OA progression.

**Conclusion:**

Despite the clear need for biomarkers of OA, the lack of coordination in current research has led to incompatible results. As such, there is yet to be a suitable biomarker to be used in a clinical setting.

## Introduction

Osteoarthritis (OA) is one of the world’s most prevalent diseases. In the United Kingdom, 10.9% and 18.2% of the population are estimated to be affected by hip and knee OA, respectively.^1^ Despite the early onset of symptoms, there has been no widely accepted intervention for altering disease progression, and the nonsurgical treatments for OA are largely based on symptom relief. From a pathophysiological perspective, both microscopically and macroscopically, OA is a highly heterogeneous disease. This heterogeneity makes it difficult to formulate diagnostic and classification criteria for OA. Diagnostic criteria must be sufficiently broad to incorporate all phenotypes, but accurate enough to only identify people with the disease. Clinically, OA is diagnosed through a clinical history and physical examination. When subjects are being enrolled in a research study, OA is diagnosed and classified radiographically, mostly through the use of the Kellgren-Lawrence (K-L) framework. The K-L framework is based on subjective analyses and thus predisposes the results to observer bias. It is also not analogous to pain and function of the patient. For this reason, it has been postulated that measurable molecular biomarkers could provide a novel, and more objective method for diagnosing and monitoring treatment effects in patients with OA.

A biomarker is defined as “a characteristic that is objectively measured and evaluated as an indicator of normal biologic processes, pathogenic processes, or pharmacologic responses to a therapeutic intervention.”^2^ In 2006, Bauer *et al*.^3^ proposed the BIPED system for classifying molecular and genetic biomarkers in OA. The acronym stands for; B—burden of disease, I—Investigative, P—prognostic, E—efficacy of intervention, D—diagnostic. In 2011, another category was added—safety (S). This classification system was designed to help direct research into biomarkers for use in clinical trials. Having an objective method of staging, predicting disease progression and identifying OA patients would be an invaluable asset in a clinical environment.

A comprehensive review of biomarker research was published in 2013 following a meeting of the European Society for Clinical and Economic Aspects of Osteoporosis and Osteoarthritis (ESCEO). It was concluded that no biomarker investigated had shown sufficient evidence to guide clinical trials or be used in a clinical environment. The review included a description of areas requiring further research and development to facilitate the use of biomarkers in OA.^4^ This systematic review aims to provide an up-to-date list and analysis of molecular biomarkers in knee OA.

## Methods

### Search Strategy

A literature search was performed on 2 electronic databases: MEDLINE (1946 to April 2018) and Embase (1974 to April 2018). These databases were selected so as to remain consistent with the ESCEO review. The terms “knee osteoarthritis,” “hip osteoarthritis,” and “osteoarthritis” were combined using the “OR” function. These terms were then combined with “biomarker” using the “AND” function. All subheadings were included for each of the search terms.

Results were then limited to human studies in the past 5 years. These results were reviewed by title and abstract using the inclusion and exclusion criteria (Table 1).

**Table 1.** Inclusion and Exclusion Criteria When Reviewing Studies for Inclusion in the Systematic Review.

Inclusion criteria	1. Study conducted after August 2013 2. Human study 3. Study in the English language 4. Study provided data regarding knee OA 5. Study focused on molecular biomarkers
Exclusion criteria	1. Study focused on patients with secondary OA 2. Study tested therapeutic interventions 3. Abstract-only publications 4. Study focused on genetic, cellular, or imaging biomarkers 5. Review articles 6. Studies graded “poor” according to the NIH Study Quality Assessment Tool

NIH =- National Institutes of Health; OA = osteoarthritis.

### Assessment of Study Quality

Quality of the studies was assessed by one reviewer using the National Institutes of Health (NIH) Study Quality Assessment Tool. This tool had subsections that were applicable for assessing meta-analyses and case-control, cohort, and cross-sectional studies. It uses a series of questions to help the user asses the internal validity of a study and to what extent the results of the study can be considered valid.^5^ The total number of yes’ is then interpreted to give an overall quality rating for the study. For cohort and cross-sectional studies, there are 14 questions: 0 to 4, poor study; 5 to 9, fair study; and 10 to 14, good study. For meta-analyses, there are 8 questions: 0 to 2, poor study; 3 to 5, fair study; and 6 to 8, good study. For case-control studies, there are 12 questions: 0 to 4, poor study; 5 to 8, fair study; and 9 to 12, good study.

### Data Extraction

Data extracted from the studies included the markers studied, the BIPEDS class investigated for the biomarker, source of biomarker, biomarker analysis method and statistical data, including *P*-values, odds ratios (OR), and correlation coefficients, as appropriate. Statistical significance was set at *P* = 0.05.

### Data Presentation

Initially the molecular biomarkers identified in the studies were categorized into 4 broad subgroups: matrix degrading enzymes, matrix molecules, regulatory molecules, and other molecules. The 4 groups are presented in individual tables in this review. Relevant statistical information to support/oppose the BIPEDS classification is included in the tables. A further table listing the algorithms identified in the studies is also presented in this review. Studies that found no significant connection between the marker and OA are included in Supplementary Appendix 1.

## Results

### Literature Search

**Figure 1.**
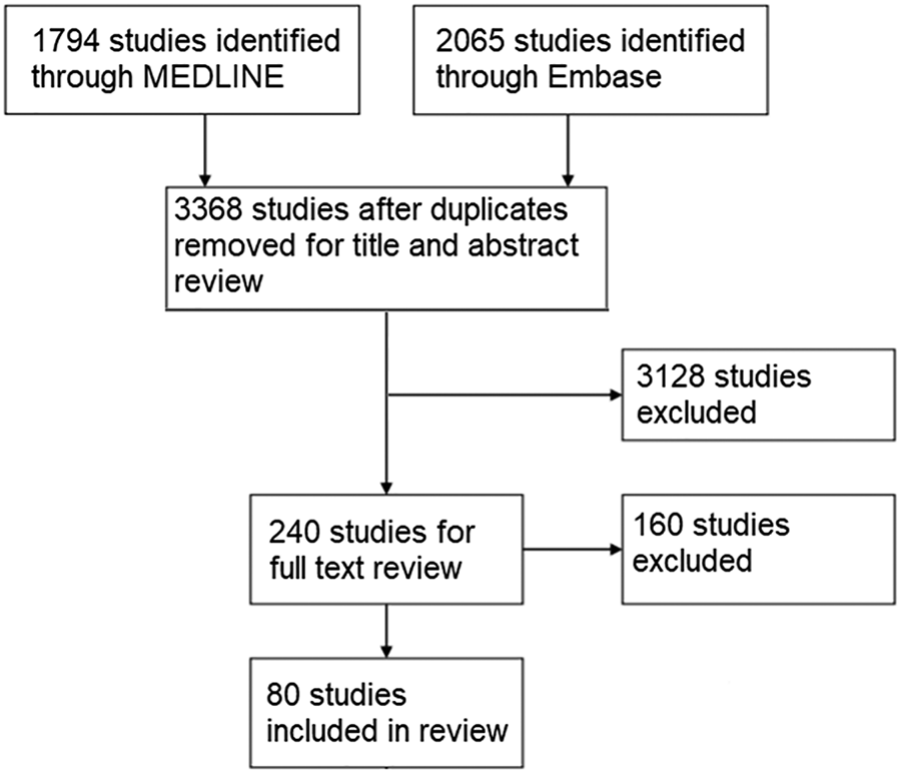
Flowchart of study selection.

A total of 80 studies were identified in total that fit the inclusion and exclusion criteria to be included in the review (Figure 1). The NIH score for each study along with biomarker source and method of laboratory analysis are listed in Supplementary Appendix 2.

### Matrix Degrading Enzymes

Eight molecules were identified that were appropriate for this category (Table 2). With regard to the BIPEDS method, 9 were investigated as burden of disease markers and 6 as diagnostic markers. Li *et al*.^6^ provided evidence for a disintegrin and metalloproteinase with thrombospondin motifs (ADAMTS)-4 and ADAMTS-5 that demonstrated they were present in significantly different concentrations in early osteoarthritis (eOA) than in later stages of OA in serum. Matrix metalloproteinase (MMP)-1 and MMP-3, the most studied marker in this category, were shown to be significantly elevated in OA patients compared with healthy subjects and eOA patients. MMP-3 also had an area under the curve (AUC) value of 0.690 when a receiver operating characteristic (ROC) analysis was carried out for its diagnostic ability. In this study, eOA patients were defined as Kellgren-Lawrence (K-L) grade 1/2.^6^

**Table 2.** Degradation Enzymes With Statistically Significant Association to Knee Osteoarthritis.^a^

Marker	Study	Test Subject (*N*)	Control (*N*)	BIPEDS Class	Statistical Association
ADAMTS-4	^6^	eOA 44, iOA and aOA 26	30	B, D	S eOA > iOA and aOA (*P* < 0.01)
ADAMTS-5	^6^	eOA 44, iOA and aOA 26	30	B, D	S iOA and aOA > eOA and HC (*P* < 0.01)
Autotaxin	^24^	70	20	B, D	**S OA > HC (*P* = 0.02)** S conc. ∝ K-L grade (*r* = 0.776, *P* < 0.001) S conc. ∝ WOMAC score (*r* = 0.558, *P* < 0.001) SF OA > HC (*P* = 0.01) SF conc. ∝ K-L grade (*r* = 0.606, *P* < 0.001) SF conc. ∝ WOMAC score (*r* = 0.371, *P* = 0.002) SF conc. ∝ S (*r* = 0.639, *P* < 0.001)
MMP-1	^6^	eOA 44, iOA and aOA 26	30	B, D	S iOA and aOA > eOA and HC (*P* < 0.05)
MMP-3	^6^	eOA 44, iOA and aOA 26	30	B, D	S iOA and aOA > eOA and HC (*P* < 0.05)
	^25^	56	31	B, D	S OA > HC (*P* = 0.003) Diagnostic: AUC = 0.690, Sen = 0.750, Spec = 0.613 at 26.65 ng/mL Discriminating isolated KOA from generalized OA: AUC = 0.822, Sen = 0.731, Spec = 0.869 at 41.35 ng/ml
MMP-13	^26^	34	52	B	S conc. ∝ length of LCD (*r* = 0.48, *P* = 0.02)
TRAcP5b	^27^	181	16	B	Baseline S conc. ∝ WOMAC pain score (β = 1.28, *P* = 0.02) Baseline S conc. ∝ subchondral sclerosis (β = 0.35, *P* = 0.02) Baseline S conc. ∝ NHANES 1 pain change (*P* = 0.008)
TG2	^28^	OA 19, OA + OP 15	5	B	Bo OA > HC (*P* < 0.01)

ADAMTS = A disintegrin and metalloproteinase with thrombospondin motifs; aOA = advanced osteoarthritis; AUC = area under curve; B = burden of disease; Bo = Bone; D = diagnostic; eOA = early osteoarthritis; HC = healthy control; iOA = intermediate osteoarthritis; KL = Kellgren-Lawrence; KOA = knee osteoarthritis; MMP = matrix metalloproteinase; NHANES = National Health and Nutrition Examination Survey; OA = osteoarthritis; *r* = Pearson’s correlation coefficient; S = serum; sen = sensitivity; SF = synovial fluid; spec = specificity; TG2 = tissue transglutaminase 2; TRAcP5b = tartrate-resistant acid phosphatase 5b; WOMAC = Western Ontario and McMaster Universities Osteoarthritis Index; β = beta coefficient.

a Boldfaced text highlights consistencies between biomarker sources.

### Matrix Molecules

A total of 21 markers were grouped as matrix molecules (Table 3); 20 were investigated as burden of disease markers, 17 as diagnostic markers and 15 as prognostic markers. The Foundation for the National Institutes of Health (FNIH) OA biomarkers consortium evaluated the ability of 14 biomarkers in serum, urine or both to predict case status at 48 months and differentiate between 3 progressor types; pain progression, joint space loss progression and pain and joint space loss progression over 48 months. Twelve- and 24-month time integrated concentrations (TICs) of urinary Col2-3/4 C-terminal cleavage product of human type II collagen (C2C) predicted progression in all 3 progressor types.^7^ C-telopeptide of type II collagen (CTXII) was shown to have the best predictive ability of case status and progression. CTXII was the most studied biomarker from all 4 groups. With regard to knee OA progression both serum and urine CTXII concentrations were shown to predict this.^8,9^ Despite being investigated by 11 studies this was the only parameter that was investigated and reported as being statistically significant in 2 sources. Kraus *et al*.^7^ showed the ability of urinary and serum NTX-1 concentrations at 12 and 24 months to predict 48-month case status. Using K-L grade to define OA, He *et al*.^10^ reported a significant difference in C-Col10 between K-L grade 0 and K-L grade 2 (*P* = 0.04). Serum concentrations of hyaluronic acid (HA) were correlated with progression of joint space narrowing in patients classified as K-L grade 0/1 (β = 0.15, *P* = 0.021).^11^

**Table 3.** Matrix Molecules with Statistically Significant Association to Knee Osteoarthritis.^a^

Marker	Study	Test Subject (*N*)	Control *N*	BIPEDS Class	Statistical Association
C1M	^29^	342	—	B	S: TKR > moderate OA (*P* < 0.001) TKR > severe OA (*P* = 0.003)
	^30^	TKR 104	—	B	S conc. ∝ periligamentous synovitis (β=0.012, p=0.18)
	^31^	58	33	B	S OA > HC (*P* < 0.02)
C2C	^7^	194	406	B, P, D	U: 24M TIC associated with 48M case status (OR = 1.27, *P* = 0.0108) Baseline associated with JSL and pain progression (OR = 1.27, *P* < 0.05) 12M TIC associated with JSL and pain progression (OR = 1.35, *P* < 0.05) 24M TIC associated with JSL and pain progression (OR = 1.50, *P* < 0.05)
C2M	^29^	342	—	B	S: TKR > severe OA (*P* < 0.001) TKR > moderate OA (*P* < 0.001)
	^32^	252	279	B, D	SF conc. ∝ K-L grades (*r*_s_ = 0.842, *P* < 0.001)
	^8^	920	2505	B, P	S conc. associated with KOA incidence (OR = 1.95, *P* = 0.0117) S conc. associated with KOA progression (OR = 1.69, *P* = 0.0311)
	^33^	216	64	D	S OA > HC (*P* = 0.019)
C3M	^33^	216	64	D	S OA > HC (*P* = 0.019)
	^31^	58	33	B	S OA < HC (*P* < 0.03)
C-Col10	^10^	261	10	B, D	S: K-L grade 0 < K-L grade 2 (*P* = 0.04) C-Col10 ∝ C2M (*r* = 0.545, *P* < 0.0001) C-Col10 ∝ hsCRP (*r* = 0.233, *P* < 0.0001)
Coll2-1NO2	^34^	254	—	B, P	Baseline U conc. associated with incident KOA (OR = 0.74, *P* < 0.05)
COMP	^25^	56	31	B, D	S: OA > HC (*P* < 0.001) Diagnostic: AUC= 0.757, Sen = 0.732, Spec = 0.710 at 717.5 ng/mL
	^18^	594	394	P, D	Baseline S conc. associated with OA incidence (OR = 1.3, *P* = 0.03) Baseline S conc. associated with progression (OR = 1.2, *P* = 0.03)
	^8^	920	2505	B, P	S conc. associated with KOA incidence (OR = 5.85, *P* < 0.002) S conc. associated with KOA progression (OR = 4.07, *P* < 0.002) S conc. associated with KOA risk (OR = 3.26, *P* < 0.002) S conc. associated with KOA severity (OR = 1.45, *P* < 0.002)
	^35^	594	394	B	S conc. ∝ OP area in painful knees only (β = 0.092, *P* = 0.005)
	^9^	723	—	B, P, D	S conc. associated with KOA incidence (OR = 0.725, *P* = 0.016) S conc. associated with KOA progression (OR = 1.496, *P* = 0.006)
CRPM	^29^	342	—	B	S TKR > moderate OA (*P* = 0.003)
	^18^	1335	—	P, D	Baseline S conc. associated with progression (OR = 1.3, *P* = 0.01)
	^36^	281	64	B	S conc. ∝ conditioning pain modulation (*P* < 0.05) S conc. ∝ temporal summation (*P* < 0.05)
	^33^	216	64	D	S OA > HC (*P* = 0.04)
CS846	^9^	723	—	B, P, D	S conc. associated with KOA presence (OR = 1.273, *P* = 0.005)
CTX-I	^7^	194	406	B, P, D	S: 12M TIC associated with 48M case status (OR = 1.29, *P* = 0.0057) 24M TIC associated with 48M case status (OR = 1.28, *P* = 0.0051) Baseline associated with JSL and pain progression (OR = 1.24, *P* < 0.05) 12M TIC associated with JSL and pain progression (OR = 1.36, *P* < 0.05) 24M TIC associated with JSL and pain progression (OR = 1.34, *P* < 0.05)
	^35^	594	394	B	U conc. ∝ Min JSW (β = −0.128, *P* = 0.001) U conc. ∝ OP area (β = −0.072, *P* = 0.029)
	^9^	723	—	B, P, D	U conc. associated with knee OA presence (OR = 1.232, *P* = 0.022)
	^37^	600	—	B, P	S conc. associated with MOAKS grade 3 and 4/5 subregions BMLs (*P* < 0.05)
CTX-Iα	^7^	194	406	B, P, D	U: Baseline associated with 48M case status (OR = 1.20, *P* = 0.0364) 12M TIC associated with 48M case status (OR = 1.28, *P* = 0.0065) 24M TIC associated with 48M case status (OR = 1.32, *P* = 0.0020) 12M TIC associated with JSL and pain progression (OR = 1.34, *P* < 0.05) 24M TIC associated with JSL and pain progression (OR = 1.39, *P* < 0.05)
	^37^	600	—	B, P	U conc. associated with MOAKS 4/5 sub-regions BMLs (p<0.05)
CTX-Iβ	^7^	194	406	B, P, D	U: 12M TIC associated with 48M case status (OR = 1.27, *P* < 0.01) 24M TIC associated with 48M case status (OR = 1.27, *P* < 0.001) 12M TIC associated with JSL and pain progression (OR = 1.32, *P* < 0.05) 24M TIC associated with JSL and pain progression (OR = 1.35, *P* < 0.05)
	^37^	600	—	B, P	U conc. associated with MOAKS grade 3 size and 4/5 subregions BMLs (*P* < 0.05)
CTX-II	^7^	194	406	B, P, D	U: Baseline conc. associated with 48M case status (OR = 1.29, *P* = 0.0049) 12M TIC associated with 48M case status (OR = 1.35, *P* = 0.0015) 24M TIC associated with 48M case status (OR = 1.37, *P* = 0.0006) Baseline, 12M, 24M TIC associated with JSL/Pain/JSL+pain progression (1.48 < OR < 1.72, *P* < 0.05)
	^18^	1335	—	P, D	S conc. associated with KOA progression (OR = 1.3, *P* = 0.01)
	^8^	920	2505	B, P	S conc. associated with KOA incidence (OR = 1.74, *P* < 0.002) S conc. associated with KOA progression (OR = 2.73, *P* < 0.002) S conc. associated with KOA risk (OR = 5.72, *P* < 0.002) S conc. associated with KOA severity (OR = 5.72, *P* < 0.002)
	^35^	594	394	B	U conc. ∝ Min JSW (β = −0.107, *P* = 0.004) U conc. ∝ OP area in painful knees only (β = 0.062, *P* = 0.044)
	^9^	723	—	B, P, D	U conc. associated with KOA presence (OR = 1.264, *P* = 0.010) U conc. associated with KOA progression (OR = 1.653, *P* = 0.001)
	^37^	600	—	B, P	U conc. associated with MOAKS grade 2 size BMLs and 4/5 subregions BMLs (*P* < 0.01) Predicting BML presence: AUC = 0.688
	^38^	149	—	B, P	U conc. ∝ total osteophytes (*r* = 0.336, *P* = 0.008) Baseline: Progressors > Nonprogressors (*P* = 0.004) U conc. associated with osteophyte progression (RR = 5.4, *P* < 0.0001)
	^39^	140	—	B, P, D	U conc. associated with medial tibiofemoral cartilage defects (OR = 4.36, *P* = 0.004) U conc. associated with lateral tibiofemoral BMLs (OR = 10.62, *P* = 0.01)
	^40^	82	20	B, D	U OA > HC (*P* < 0.001)
	^41^	472	517	B, D	U OA > HC (*P* = 0.001)
	^42^	424	616	B, D	U: K-L grade 3/4 > K-L grade 1/2 in men (*P* < 0.01) K-L grade 3/4 > K-L grade 1/2 in women (*P* < 0.01) K-L grade 2 > K-L grade 1 in women (*P* < 0.01) Diagnosing radiological OA: AUC = 0.5229
Fib3-1	^43^	241	—	P	S conc. associated with ACR-criteria (OR = 2.70, *P* < 0.05) S conc. associated with chronic pain (OR = 2.62, *P* < 0.05)
HA	^7^	194	406	B, P, D	S: 24M TIC associated with 48M case status (OR = 1.22, *P* < 0.05) 12M TIC associated with JSL and pain progression (OR = 1.29, *P* < 0.05) 24M TIC associated JSL and pain progression (OR = 1.34, *P* < 0.05)
	^11^	444	—	B, P	S conc. ∝ Progression from K-L grades 2/3 (β = 0.02, *P* = 0.004) S conc. ∝ Progression of JSN in K-L 0/1 (β = 0.15, *P* = 0.021) S conc. ∝ Progression of JSN in K-L 2/3 (β = 0.24, *P* = 0.008)
	^35^	594	394	B	S conc. ∝ Min JSW (β = 0.100, *P* = 0.022) S conc. ∝ OP area in painful knees (β = 0.116, *P* = 0.004)
hsCRP	^24^	70	20	B, D	S OA > HC (*P* < 0.001)
	^29^	342	—	B	S TKR > mild OA (*P* = 0.003) S TKR > moderate OA (*P* < 0.001)
	^44^	6440	10,650	B, D	S OA > HC (*P* < 0.001)
	^31^	58	33	B	S OA > HC (*P* < 0.02)
NTX-1	^7^	194	406	B, P, D	**S 12M TIC associated with 48M case status (OR = 1.28, *P* = 0.0064)** S 24M TIC associated with 48M case status (OR = 1.25, *P* = 0.0131) U 12M TIC associated with 48M case status (OR = 1.24, *P* = 0.0199) U 24M TIC associated 48M case status (OR = 1.29, *P* = 0.0057) S 12M TIC associated with JSL and pain progression (OR = 1.29, *P* < 0.05) S 24M TIC associated with JSL and pain progression (OR = 1.27, *P* < 0.05) U 12M TIC associated with JSL and pain progression (OR = 1.31, p<0.05) U 24M TIC associated with JSL and pain progression (OR = 1.37, *P* < 0.05)
	^35^	594	394	B	U conc. ∝ Min JSW (β = −0.130, *P* < 0.001) U conc. ∝ OP area (β = −0.077, *P* = 0.017)
	^37^	600	—	B, P	U conc. associated with 4/5 MOAKS subregions BMLs (*P* < 0.05)
OC	^9^	723	—	B, P, D	S conc. associated with knee OA presence in painful knees (OR = 1.281, *P* = 0.016)
PIIANP	^7^	194	406	B, P, D	S 12M TIC associated with 48M case status (OR = 0.83, *P* < 0.05)
	^9^	723		B, P, D	S conc. associated with KOA presence (OR = 1.300, *P* = 0.003)
PIIINP	^35^	594	394	B	S conc. ∝ Min JSW (β = 0.122, *P* = 0.002)
ucMGP	^45^	178	160	B	S OA < HC (*P* = 0.045) SF conc. ∝ K-L grades (*r*_s_ = −0.479, *P* < 0.001)

ACR = American College of Rheumatology; B = burden of disease; BML = bone marrow lesion; C1M = MMP-mediated degradation of type I collagen, C2C- Col2-3/4 C-terminal cleavage product of human type II collagen; C2M = MMP-mediated degradation of type 2 collagen; C3M = MMP-mediated degradation of type 3 collagen; C-Col10 = C-terminus of collagen X; Coll2-1 NO2 = nitrated epitope of the α-helical region of type II collagen; COMP = cartilage oligomeric matrix protein; CRPM = matrix metalloproteinase-dependent degradation of C-reactive protein; CS846 = chondroitin sulfate epitope 846; CTXI = C-terminal cross-linked telopeptide type I collagen; CTXII = C-terminal cross-linked telopeptide type II collagen; D = diagnostic; Fib = fibulin; HA = hyaluronic acid/hyaluronan; HC = healthy control; hsCRP = high sensitivity C-reactive protein; JSL = joint space lesion; JSW = joint space width; K-L = Kellgren-Lawrence; KOA = knee osteoarthritis; M = months; MOAKS = MRI Osteoarthritis Knee Score; NTXI = cross-linked N-telopeptide of type I collagen; OA = osteoarthritis; OC = osteocalcin; OP = osteophyte; OR = odds ratio; P = prognostic; PIIANP = N-terminal pro-peptide of collagen IIA; PIIINP = N-terminal pro-peptide of procollagen type III; *r* = Pearson’s correlation coefficient; *r*_s_ = Spearman’s correlation coefficient; S = serum; sen = sensitivity; SF = synovial fluid; spec = specificity; TIC = time integrated concentration; TKR = total knee replacement; U = urine; ucMGP = uncarboxylated matrix Gla protein; β = beta coefficient.

a Boldfaced text highlights consistencies between biomarker sources.

### Regulatory Molecules

A total of 35 regulatory markers were identified in the studies (Table 4). With regard to the BIPEDS method, 33 were investigated as burden of disease markers, 21 as diagnostic markers and 6 as prognostic markers. β-catenin was significantly reduced in eOA compared with late/intermediate stage OA (*P* < 0.05). The same study also demonstrated that serum concentrations of transcription factor 4 were significantly higher in eOA patients when compared with healthy controls (*P* < 0.002). Classification of stage of OA was carried out for 32 patients using the Mankin scoring system following a TKR.^12^ Indian hedgehog (IHh) protein was elevated in SF in eOA patients, classified as patients with Outerbridge scale 1/2 cartilage breakdown, compared with healthy controls (*P* < 0.001).^13^ Using K-L grades 1/2 as a definition of eOA, serum concentrations of angiopoietin-2, IL-8, follistatin, granulocyte-colony stimulating factor (G-CSF), vascular endothelial growth factor (VEGF), and hepatocyte growth factor were shown to be significantly different in eOA than in HCs.^14^ Synovial fluid and serum concentration of VEGF have also been reported as being correlated with K-L grade in 2 separate studies.^14,15^

**Table 4.** Regulatory Molecules with Statistically Significant Association to Knee Osteoarthritis.^a^.

Marker	Study	Test Subjects (*N*)	Control (*N*)	BIPEDS Class	Statistical Association
Adiponectin	^46^	60	25	B, D	S conc. ∝ WOMAC total (*r*_s_ = 0.477, *P* = 0.001) S conc. ∝ 1/SF-36 bodily pain (*r*_s_ = −0.373, *P* = 0.03) S conc. ∝ K-L grades (*r*_s_ = 0.572, *P* < 0.001)
	^47^	138	—	P	S conc. ∝ Global femur CV (*r*_s_ = 0.17, *P* = 0.041)
	^48^	OA 19, 15 OA + OP 15	5	B, D	SF OA > HC (*P* < 0.002)
Adipsin	^47^	138	—	P	S conc. ∝ Global knee CV (*r*_s_ = −0.28, *P* = 0.001)
Adropin	^49^	60	30	B, D	S: KOA < HC (*P* < 0.001) K-L grade 4 > K-L grade 1/2/3 (*P* < 0.01)
Angiopoietin-2	^14^	31	15	B, D	S KOA > HC (*P* < 0.001) S conc. > SF conc. (*P* < 0.001) S eOA > HC (*P* < 0.001) S aOA > eOA (*P* = 0.02)
β-catenin	^12^	TKR 32 Tibial plateau samples 45 [early 5, intermediate 13, late 17]	—	B	Subchondral Bo ∝ Mankin score (*r* = 0.775, *P* < 0.01) Trabecular Bo ∝ Mankin score (*r* = 0.769, *P* < 0.01) Bo Late/intermediate stage OA > eOA (*P* < 0.05)
BMP-2	^50^	37	20	B, D	S OA > HC (*P* < 0.001) S K-L grade 4 > K-L grade 2/3 (*P* < 0.001) **S conc. ∝ K-L grades (*r*_s_ = 0.81, *P* < 0.001)** S conc. ∝ WOMAC scores (*r*_s_ = 0.63, *P* < 0.001) **SF conc. ∝ K-L grades (*r*_s_ = 0.78, *P* < 0.001)** SF conc. ∝ WOMAC scores (*r*_s_ = 0.77, *P* < 0.001) SF > S (*P* < 0.001)
BDNF	^51^	27	19	B, D	S OA > HC (*P* = 0.03) S ∝ WOMAC (*r*_s_ = 0.39, *P* = 0.04)
CCL2	^52^	161	138	B, D	S OA > HC (*P* = 0.025) SF conc. ∝ Total WOMAC score (*r*_s_ = 0.460, *P* < 0.001)
CGRP	^53^	65	21	B, D	S OA > HC (*P* = 0.006) S > SF (*P* < 0.001) S conc. ∝ SF (*r*_s_ = 0.606, *P* < 0.001) **S conc. ∝ K-L grades (*r*_s_ = 0.565, *P* < 0.001)** S conc. ∝ Total WOMAC score (*r*_s_ = 0.554, *P* < 0.001) **SF conc. ∝ K-L grades (*r*_s_ = 0.441, *P* < 0.001)** SF conc. ∝ Total WOMAC score (*r*_s_ = 0.426, *P* < 0.001)
CXCL12	^54^	244	244	B	S OA > HC (*P* < 0.001) S Diagnosis: AUC = 0.850, sen = 0.784, spec = 0.802 at 5.5 ng/mL S conc. ∝ K-L grade (*r*_s_ = 0.461, *P* < 0.0001) S conc. ∝ SF conc. (*r*_s_ = 0.344, *P* < 0.0001) **S Active OA: AUC 0.839, sen = 0.784, spec = 0.802 at 10.5 ng/mL** **SF Active OA: AUC = 0.855, sen = 0.813, spec = 0.784 at 15.0 ng/mL**
DKK-1	^55^	40	20	B, D	SF diagnosis: AUC = 0.801, sen = 0.68, spec = 0.92 at 88.2 pg/mL **S Predicting K-L grade 4 OA: AUC = 0.684, sen = 0.91, spec = 0.56 at 1100 pg/mL** **SF predicting K-L grade 4 OA: AUC = 0.745, sen = 0.82, spec = 0.72 at 104 pg/mL**
FGF-23	^56^	50	20	B	KOA > HC (*P* = 0.023) S conc. ∝ WOMAC (*r* = 0.364, *P* = 0.009) S conc. ∝ K-L grade (*r* = 0.355, *P* = 0.011)
Follistatin	^14^	31	15	B, D	S KOA > HC (*P* < 0.001) S > SF (*P* < 0.001) S conc. ∝ 1/K-L grade (*r*_s_ = −0.374, *P* < 0.05) S eOA > HC (*P* < 0.001)
Fractalkine	^57^	193	182	B	S conc. ∝ WOMAC total score (*r* = 0.405, *P* = 0.025) **S conc. ∝ K-L grade (*r* = 0.542, *P* < 0.001)** SF conc. ∝ WOMAC total score (*r* = 0.359, *P* = 0.02) **SF conc. ∝ K-L grade (*r* = 0.563, *P* < 0.001)**
G-CSF	^14^	31	15	B, D	S KOA > HC (*P* < 0.001) S > SF (*P* < 0.001) S eOA > HC (*P* < 0.001)
Gremlin-1	^58^	212	125	B, D	S KOA > HC (*P* < 0.001) **S conc. ∝ K-L grade (*r*_s_ = 0.372, *P* < 0.001)** **SF conc. ∝ K-L grade (r_s_ = 0.483, *P* < 0.001)**
HGF	^14^	31	15	B, D	S KOA > HC (*P* < 0.001) S > SF (*P* < 0.001) S eOA > HC (*P* < 0.001)
HIF-1α	^59^	278	203	B	S KOA > HC (*P* < 0.001) S conc. ∝ K-L grade (*r*_s_ = 0.258, *P* < 0.001) SF conc. ∝ K-L grade (*r*_s_ = 0.437, *P* < 0.001)
HIF-2α	^60^	TKR 29, arthroscopy- 16	Traumatic knee 5	B	C KOA > HC (*P* < 0.05) C conc. ∝ K-L grade (*r* = 0.7001, *P* < 0.05)
IL1Ra	^61^	146	—	B, P	S: Predictivity for baseline radiographic severity: Baseline AUC = 0.59, *P* = 0.042; 18M AUC = 0.67, *P* = 0.001 Predictivity for 24M radiographic severity: 18M AUC = 0.69, *P* = 0.004 Baseline conc. ∝ medial JSN at 24M (*r* = 0.193, *P* = 0.047)
IL-6	^30^	aOA 104	—	B	SF conc. ∝ parapatellar synovitis (β = 0.006, *P* = 0.03) SF conc. ∝ Synovial volume (β = 0.040, *P* = 0.030)
	^62^	160	—	B	S conc. ∝ Pain VAS score in eOA (β = 10.77, *P* < 0.01) S conc. ∝ JKOM-pain score in eOA (β = 3.19, *P* < 0.001)
	^63^	53	48	B	S KOA > HC (*P* = 0.031) S conc. ∝ VAS score (*r*_s_ = 0.238, *P* = 0.017) S conc. ∝ WOMAC pain (*r*_s_ = 0.251, *P* = 0.012) S conc. ∝ WOMAC rigidity (*r*_s_ = 0.303, *P* = 0.002)
IL-8	^14^	31	15	B, D	S KOA > HC (*P* < 0.001) S eOA > HC (*P* < 0.001)
IL-10	^63^	53	48	B	S KOA > HC (*P* = 0.030) S conc. ∝ VAS score (*r*_s_ = 0.203, *P* = 0.042) S conc. ∝ WOMAC rigidity (*r*_s_ = 0.271, *P* = 0.007) S conc. ∝ WOMAC rigidity (*r*_s_ = 0.271, *P* = 0.007)
IL-17	^64^	226	106	B	SF conc. ∝ WOMAC pain (*r* = 0.279, *P* < 0.05)
	^65^	194	—	B	S associated with lateral tibial cartilage defects (OR = 1.40, *P* = 0.023) S associated with patellar cartilage defects (OR = 1.33, *P* = 0.029)
Indian hedgehog	^13^	TKR 40	80	B, D	**SF eOA > HC (*P* < 0.001)** SF eOA conc. ∝ normal/early Outerbridge score (*r*_s_ = 0.556, *P* < 0.001) C OA > HC (*P* < 0.001)
Leptin	^14^	31	15	B, D	S conc. ∝ SF (*r*_s_ = 0.876, *P* < 0.001)
	^47^	138		P	S conc. ∝ Global knee CV (*r* = −0.23, *P* = 0.006)
	^26^	34	52	B	S conc. ∝ length of medial osteophytes (*r* = 0.55, *P* = 0.006) S conc. ∝ length of medial capsular distension (*r* = 0.55, *P* = 0.007) SF conc. ∝ depth of effusion (*r* = −0.46, *P* = 0.01)
PGE2	^66^	291	58	B, P, D	SKOA > HC (*P* < 0.0001)
PRDX6	^65^	194	—	B	S associated with lateral femoral cartilage defects (OR = 1.23, *P* = 0.001) S associated with lateral tibial cartilage defects (OR = 1.14, *P* = 0.003) S associated with medial tibial cartilage defects (OR = 1.13, *P* = 0.006) S associated with lateral femoral cartilage defects (OR = 1.10, *P* = 0.027) S associated with lateral femoral BMLs (OR = 1.19, *P* < 0.001) S associated with lateral tibial BMLs (OR = 1.13, *P* = 0.014)
	^67^	74	79	B	S KOA > HC (*P* = 0.035) SF conc. ∝ K-L grades (*r* = 0.642, *P* < 0.001) SF conc. ∝ Total WOMAC score (*r* = 0.491, *P* < 0.001) SF conc. ∝ Noyes score (*r* = 0.543, *P* < 0.001)
Sclerostin	^12^	TKR 32 Tibial plateau samples 45 [early 5, intermediate 13, late 17]		B	Subchondral Bo conc. ∝ 1/Mankin score (*r* = −0.632, *P* < 0.01) Trabecular Bo conc. ∝ 1/Mankin score (*r* = −0.620, *P* < 0.01) Bo aOA/iOA < eOA (*P* < 0.05)
	^68^	95	95	B, D	S OA < HC (*P* = 0.004) S > SF (*P* < 0.001) S conc. ∝ SF (*r* = 0.657, *P* < 0.001) **S conc. ∝ 1/K-L grades (*r* = −0.464, *P* < 0.001)** **SF conc. ∝ 1/K-L grades (*r* = −0.592, *P* < 0.001)**
TGF-β1	^69^	160	80	B, D	S: OA > HC (*P* < 0.05) K-L 2, 3 and 4 > K-L 1 (*P* = 0.002) Diagnostic: AUC = 0.889, Sen = 0.71, Spec = 0.90 at 12.11 ng/mL
TNF-α	^70^	51		B	SF conc. ∝ K-L grade (β = 2.92, *P* = 0.03)
	^49^	60	30	B, D	S OA > HC (*P* < 0.001)
Transcription factor 4	^12^	TKR 32 Tibial plateau samples 45 [early 5, intermediate 13, late 17]		B	Bo aOA/iOA > eOA (*P* < 0.05)
TSG-6	^71^	132	—	P	SF: TKR > Non-TKR (*P* = 0.002) Non-progressor > Progressor (*P* < 0.0001) Progression to TKR: AUC = 0.90, sen = 0.91, spec = 0.82 at 13.3 U/mL
VEGF	^14^	31	15	B, D	S KOA > HC (*P* < 0.001) S < SF (*P* < 0.001) SF conc. ∝ K-L grade (*r*_s_ = 0.367, *P* = 0.04) S eOA > HC (*P* < 0.002)
	^60^	TKR 29, knee arthroscopy 16	Traumatic knee 5	B	C conc. ∝ K-L grades 3/4 (*r* = 0.6647, *P* < 0.05)
	^15^	80	20	B	S conc. ∝ K-L grade (*r* = 0.454, *P* < 0.001) SF conc. ∝ K-L grade (*r* = 0.727, *P* < 0.001)
	^26^	34	52	B	S conc. ∝ volume of effusion (*r* = 0.41, *P* = 0.05) S conc. ∝ lateral capsular distension (*r* = 0.6, *P* = 0.002) SF conc. ∝ KL grades (*r* = 0.43, *P* = 0.02)
YKL-40	^72^	50	40	B, D	S KOA > HC (*P* < 0.05) SF conc. ∝ S (*r*_s_ = 0.532, *P* < 0.001) SF conc. ∝ Gray scale US (*r*_s_ = 0.690, *P* < 0.001) SF conc. ∝ power Doppler US (*r*_s_ = 0.303, *P* = 0.033)

aOA = advanced osteoarthritis; AUC = area under curve; B = burden of disease; BDNF = brain-derived neurotrophic factor; BML = bone marrow lesion; BMP = bone morphogenic protein; Bo = bone; C = cartilage; CCL = chemokine (C-C motif) ligand; CGRP = calcitonin gene-related peptide; CV; cartilage volume; D = diagnostic; DKK = Dickkopf-related protein; eOA = early osteoarthritis; FGF = fibroblast growth factor; G-CSF = granulocyte-colony stimulating factor; HC = healthy control; HGF = hepatocyte growth factor; HIF = hypoxia-inducible factor; IL = interleukin, iOA = intermediate osteoarthritis; JKOM = Japanese Knee Osteoarthritis Measure; JSN = joint space narrowing; K-L = Kellgren-Lawrence; KOA = knee osteoarthritis; LECT = leukocyte cell–derived chemotaxin; NPV = negative predictive value; OP = osteophyte; OR = odds ratio; P = prognostic; PGE2 = prostaglandin E2; PPV = positive predictive value; PRDX6 = peroxiredoxin-6; Pre-X-KD = Pre-X-rays defined knee degeneration; *r* = Pearson’s correlation coefficient, *r*_s_ = Spearman’s correlation coefficient; S = serum; Sen = sensitivity; SF = synovial fluid; SF-36 = 36-item Short Form survey; Spec = specificity; TGF = transforming growth factor; TKR = total knee replacement; TNF = tumor necrosis factor; TSG-6; tumor necrosis factor–inducible gene 6; US = ultrasound; VAS = visual analogue scale; WOMAC = Western Ontario and McMaster Universities Osteoarthritis Index; X-KOA = X-rays defined knee OA, YKL-40 = chitinase-3-like protein 1; β = beta coefficient.

a Boldfaced text highlights consistencies between biomarker sources.

### Other Molecules

A total of 25 markers did not fit into the other 3 categories (Table 5); 18 were investigated as burden of disease markers, 12 as diagnostic markers and 6 as prognostic markers as per the BIPEDS method. None of the markers in this category have been verified as potential biomarker candidates by more than 1 study. Two studies investigated amino acids. The study by Chen *et al*.^16^ that investigated alanine and taurine reported an AUC = 0.928 and AUC = 0.920, respectively, when used to diagnose OA in a study sample of 67. Arginine, investigated by Zhang *et al*.^17^ had an AUC = 0.984.

**Table 5.** Other Molecules with Statistically Significant Association to Knee Osteoarthritis.^a^.

Marker	Study	Test Subjects (*N*)	Control (*N*)	BIPEDS Class	Statistical Association
15-HETE	^66^	291	58	B, P, D	SKOA > HC (*P* < 0.0001) S conc. ∝ JSN (*r* = 0.374, *P* = 0.022)
4-Hydroxy-l-proline	^16^	32	35	D	S OA > HC (*P* < 0.001) Diagnostic: AUC = 0.911, sen = 0.833 and spec = 0.867
Alanine	^16^	32	35	D	S OA > HC (*P* < 0.001) Diagnostic: AUC = 0.928, sen = 0.833 and spec = 0.90
Amyloid P	^73^	77	39	B, D	S K-L grade 4 < K-L grade 2 (*P* < 0.05)
Arginine	^17^	72	76	D	S: OA < HC (*P* < 0.0001) Diagnostic: AUC = 0.984, sen = 0.983, spec = 0.89 at 57 µmol
ARGS aggrecan	^74^	40	20	P	S: OA-TKR > non-TKR OA (*P* = 0.01) OA-TKR > HC (*P* = 0.0002)
CD14	^75^	184	—	B, P	SF < S (*P* < 0.0001) CD14 conc. ∝ CD163 (*r* = 0.59, *P* < 0.0001) SF conc. ∝ Osteophyte severity. EC20 scan cohort (β = 3.473, *P* < 0.0001), POP cohort (β = 0.686, *P* < 0.0001) SF conc. ∝ JSN severity. EC20 scan cohort (β = 0.608, *P* = 0.025), POP cohort (β = 0.098, *P* = 0.035)
CD163	^75^	184	—	B, P	SF > S (*P* = 0.009) SF conc. ∝ OP severity (β = 2.820, *P* = 0.0003) SF conc. ∝ OP progression (β = 0.056, *P* < 0.0001)
CD31/PECAM-1	^14^	31	15	B, D	S KOA > HC (*P* < 0.001) S eOA > HC (*P* = 0.01)
CD40	^76^	975	—	B, P	S conc. ∝ KOA presence (*P* < 0.01) S conc. ∝ KOA progression (*P* < 0.05)
FABP4	^77^	248	15	B, D	S conc. ∝ K-L grade (*P* = 0.005) S diagnostic: AUC = 0.68, sen = 0.48, spec = 0.93 at 15.45 ng/mL
Ghrelin	^78^	52	52	B	SF conc. ∝ 1/K-L grade (*r* = −0.591, *P* < 0.001) SF conc. ∝ 1/Lequesne index (*r* = −0.308, *P* < 0.025) SF conc. ∝ 1/VAS scores (*r* = −0.591, *P* < 0.001) SF conc. ∝ Lysholm scores (*r* = 0.381, *P* = 0.005) SF conc. ∝ 1/TNF-alpha (*r* = −0.424, *P* = 0.002) SF conc. ∝ 1/IL-6 (*r* = −0.428, *P* = 0.002) K-L grade 3 vs K-L grade 4: AUC = 0.725, *P* = 0.022
Haptoglobin	^73^	77	39	B, D	S OA > HC (*P* < 0.05)
LBP	^79^	25	—	B	S conc. ∝ OP severity (β = 0.072, *P* = 0.017)
LPS	^79^	25	—	B	**S conc. ∝ OP severity (β = 0.017, *P* = 0.030)** **SF conc. ∝ OP severity (β = 0.028, *P* = 0.001)** SF conc. ∝ JSN severity (β = 0.041, *P* < 0.001) SF conc. ∝ total WOMAC score (β = 0.177, *P* = 0.008)
NPY	^80^	100	20	B, D	SF: KOA > HC (*P* = 0.0297) SF conc. ∝ Watanabe’s pain score (*r* = 0.869, *P* < 0.05) Middle > early Koshino’s radiographic grade (*P* = 0.0163) Advanced Koshino’s radiographic grade > early Koshino’s radiographic grade (*P* = 0.0352)
ox-LDL	^81^	203	194	B	S conc. ∝ K-L score (β = 0.440, *P* < 0.001)
Periostin	^82^	90	20	B	**S conc. ∝ K-L grade (*r* = 0.537, *P* < 0.001)** **SF conc. ∝ K-L grade (*r* = 0.427, *P* < 0.001)**
Sialic acid	^83^	234	160	B	SF conc. ∝ K-L grade (*r*_s_ = 0.353, *P* < 0.001)
Taurine	^16^	32	35	D	S: OA > HC (*P* < 0.001) Diagnostic: AUC = 0.920, sen = 0.867 and spec = 0.833
Thymosin β4	^84^	216	152	B, D	S OA > HC (*P* < 0.001) S conc. ∝ K-L grade (*r*_s_ = 0.253, *P* < 0.001) SF conc. ∝ K-L grade (*r*_s_ = 0.407, *P* < 0.001)
VCAM-1	^76^	925	—	B, P	S conc. ∝ KOA presence (*P* < 0.05)
vWF	^73^	77	39	B, D	S OA > HC (*P* < 0.05)
γ-Aminobutyric acid	^16^	32	35	D	S OA > HC (*P* < 0.001) Diagnostic: AUC = 0.908, sen = 0.80 and spec = 0.933

15-HETE = hydroxyeicosatetraenoic acid; AUC = area under curve; B = burden of disease; BAALC = brain and acute leukemia cytoplasmic; CD = cluster of differentiation; D = diagnostic; FABP4 = fatty acid binding protein 4; HC = healthy control; JSN = joint space narrowing; K-L = Kellgren-Lawrence; KOA = knee osteoarthritis; LBP = lipopolysaccharide binding protein; LPS = lipopolysaccharide; OP = osteophyte; P = prognostic; *r* = Pearson’s correlation coefficient, *r*_s_ = Spearman’s correlation coefficient; S = serum; Sen = sensitivity; SF = synovial fluid; SKOA = severe knee osteoarthritis; Spec = specificity; TNF = tumor necrosis factor; VAS = visual analogue scale; VCAM = vascular cell adhesion molecule; vWF = von Willebrand factor; WOMAC = Western Ontario and McMaster Universities Osteoarthritis Index; β = beta coefficient.

a Boldfaced text highlights consistencies between biomarker sources.

### Biomarker Panels

A total of 11 biomarker panels were identified in the literature included in this study (Table 6). The source of all biomarkers for use in algorithms was either serum or urine and their use was demonstrated for predicting disease presence, severity, and progression. Saberi *et al*. ^18^ presented an algorithm that consisted of patient demographics, biomarkers, and radiological input. The algorithm was developed using patient data from the Rotterdam study cohort, which consisted of 1335 patients. In this cohort, the algorithm had an excellent ability to predict disease progression over 2.5 years (AUC = 0.872).

**Table 6.** Molecular Biomarker Panels with Statistically Significant Association to Knee Osteoarthritis.

Algorithm	Use	Study	Test Subjects (*N*)	Control (*N*)	Statistical Association
24M TICs: S HA, S NTXI, U CTXII	Predicting case status at 24M	^7^	194	406	AUC = 0.618
Baseline: U CTXII, S NTXI	Predicting case status at 24M	^7^	194	406	AUC = 0.586
S CP, S Hyp, S anti-CCP antibody, age, gender	Distinguishing between HC, eOA, eRA, and non-RA inflammatory disease	^19^	eOA 16, eRA 10, non-RA 10	16	eOA: AUC = 0.86, PPV = 0.733, NPV = 0.885, sen = 0.647, spec = 0.920
S COX-2, age, BMI, gender	Prediction of JSN >0 mm/24M	^66^	291	58	AUC = 0.65
S COX-2, age, BMI, gender	Prediction of JSN >0.2 mm/24M	^66^	291	58	AUC = 0.67
S COX-2, S IL-1β, age, BMI, gender	Prediction of JSN >0.5 mm/24M	^66^	291	58	AUC = 0.64
S CTXII, S COMP, S CRPM, S C1M, age, sex, BMI, joint pain, baseline K-L score	Incidence of OA over 2.5 years	^18^	1335	—	AUC = 0.872
S CTXII, S COMP, SCRPM, SC1M, age, sex, BMI, joint pain, baseline K-L score	Progression of OA over 2.5 years	^18^	1335	—	AUC = 0.899
CXCL12, CRP, ASO, RF (all from S)	Screening for OA	^54^	244	244	AUC = 0.912
Hyp, MetSO, DT, NFK, 3-NT, CEL, CMA, G-H1, MG-H1, 3DG-H, pentosidine (all from S)	Screening for arthritic disease (eOA, eRA, non-RA)	^20^	eOA 46, aOA 17, eRA- 45, aRA 22, non-RA 42	53	AUC = 0.99, sen = 0.92, spec = 0.91, PPV = 1.0, NPV = 1.0
Anti-CCP antibody, MetSO, DT, NFK, 3-NT, CEL, CMA, G-H1, MG-H1, 3DG-H, pentosidine (all from S)	Screening for arthritic disease (eOA, eRA, non-RA)	^20^	eOA 46, aOA 17, eRA 45, aRA 22, non-RA 42	53	AUC = 0.99, sen = 0.92, spec = 0.91, PPV = 1.0, NPV = 1.0

3DG-H = 3-deoxyglucosone-derived hydroimidazolone isomers; 3-NT = 3-nitrotyrosine; aOA = advanced osteoarthritis; aRA = advanced rheumatoid arthritis; ASO = antistreptolysin-O; AUC = area under curve; BMI = body mass index; C1M = MMP-mediated degradation of type I collagen; CCP = cyclic citrullinated peptide; CEL = carboxyethyl-lysine; CMA = carboxymethylarginine; COMP = cartilage oligomeric matrix protein; COX-2 = cyclooxygenase-2; CP = citrullinated protein; CRPM = matrix metalloproteinase-dependent degradation of C-reactive protein; CTXII = C-terminal cross-linked telopeptide type II collagen; DT = dityrosine; eOA = early osteoarthritis; eRA = early rheumatoid arthritis; G-H1 = glyoxal-derived hydroimidazolone; HA = hyaluronic acid/hyaluronan; HC = healthy control; Hyp = hydroxyproline; IL = interleukin; JSN = joint space narrowing; K-L = Kellgren-Lawrence; M = months; MetSO = methionine sulfoxide; MG-H1 = methylglyoxal-derived hydroimidazolone; NFK = N-formylkynurenine = NPV = negative predictive value; NTXI = cross-linked N-telopeptide of type I collagen; OA = osteoarthritis; PPV = positive predictive value; RA = rheumatoid arthritis; sen = sensitivity; spec = specificity; TIC = time integrated concentration.

Of the 12 algorithms described below, 2 specifically targeted the early diagnosis of OA.^19,20^ Both of the studies used the same methods of patient recruitment and sampling. To be deemed as having eOA, patients had to have new onset knee pain, normal radiographs, and Outerbridge grade I/II. The algorithm consisting of citrullinated proteins (CPs), hydroxyproline, anti-CCP antibody, age and gender had the following statistics when distinguishing eOA from healthy controls and inflammatory arthritic diseases; AUC = 0.86, positive predictive value (PPV) = 0.733, and negative predictive value (NPV) = 0.885.^19^ The second algorithm for diagnosing eOA was intended for use after an individual had been excluded from the healthy control group. It combined anti-CCP antibody with biomarkers of protein oxidation, nitration, and glycation to give an AUC of 0.98.^20^

## Discussion

Using serum and urine to detect markers is advantageous because obtaining them is relatively non-invasive and samples are readily obtained. However, they effectively sample the whole body making disease localization difficult; some biomarkers, for example, regulatory and matrix molecules, are unusable as they are neither organ nor disease specific; particularly in early disease the dilutional effects of blood and extracellular fluid make the sensitivity of detection beyond that practicable. Examination of synovial fluid has the advantage of being much more specific, and with higher biomarker concentration, but in early disease synovial fluid can be difficult to obtain. To this end, it would be pertinent for future studies that analyze synovial fluid/bone/cartilage to also consider its relationship with the marker in serum/urine. A strong correlation between the two regarding the same parameter would be invaluable for the marker’s clinical applicability going forward as it would allow the reliable use of a more easily accessible source. Many markers, such as VEGF and CTXII, have demonstrated this correlation which would suggest that they warrant further investigation.

The 4 groups used to stratify the biomarkers were chosen because they represent different therapeutic pathways for research. There is evidence that supports the use of biomarkers as therapeutic targets in the development of disease-modifying osteoarthritis drugs (DMOADs). Clinical trials have used bone morphogenetic protein-7, fibroblast growth factor, and β-nerve growth factor (β-NGF) as targets in an attempt to develop new OA drugs.^21^ Tanezumab, a monoclonal antibody against β-NGF, reduced knee pain while walking by between 45% and 62% compared with 22% by placebo.^22^

Ideally, OA would be detected before it became symptomatic so that necessary measures could be taken. However, without symptomatic osteoarthritis it is very unlikely that one would contact a clinician. Bearing in mind the relative frequency and morbidity of OA, an argument could be made for a screening program of “at risk” groups along the lines of those used to detect colorectal and breast cancer. Therefore, markers that can identify eOA patients are important for a number of reasons. Having a robust and quantitative method for classifying eOA patients would provide an adjunctive outcome measure for clinical trials to measure the efficacy of disease-modifying osteoarthritis drugs or adjunctive physical therapy. This would have significant clinical relevance in everyday practice.

IHh was studied in the SF of patients classified by the Outerbridge classification. Interestingly, the study provided evidence that IHh was elevated in eOA patients (Outerbridge 1/2) and not in the control group (Outerbridge 0) or late stage OA patients (Outerbridge 3/4).^13^ If this relationship was further investigated and shown to be significant in other independent studies then it would have positive implications for diagnosing eOA. Perhaps other biomarkers may follow the same pattern as IHh and are only dysregulated during early stages of OA.

Multiple biomarker and algorithmic approaches to investigating OA have shown promise. The algorithm consisting of CP, Hyp, anti-CCP antibody, age and gender had high specificity for diagnosing eOA.^19^ Using patient demographics within the algorithm is an efficient method of increasing the algorithm’s predictive ability. It would therefore be interesting to evaluate the predictive ability of a combination of the single eOA biomarkers identified in the review. IHh protein and IL-8 both performed well as single biomarkers so perhaps their combination along with patient demographics would create a highly sensitive and specific algorithm. Due to the heterogeneity and complexity of the disease, it is likely that an algorithm will be a more effective method for making a diagnosis.

The issues surrounding the definition of eOA will continue to prove difficult unless addressed. A convincing argument put forward by Kraus^23^ suggests that for an eOA marker to be truly effective it must represent a state of preclinical OA. Preclinical OA is the stage before OA is detectable by MRI or other sensitive imaging modalities. This is the optimum time for identification from both a clinical and research perspective as it would allow early lifestyle changes and a better understanding of the efficacy of potential DMOADs, respectively. Discovery of such a marker would require a time-consuming and likely expensive follow-up of a large cohort of people if primary OA was to be the indicator. However, using patients that have experienced a knee injury and that are likely to develop secondary OA over the next 10 to 15 years may provide a solution.

In the future, development of a universal criterion for diagnosing OA to standardize recruitment in clinical trials would be extremely helpful. A universal consensus of nomenclature will help to add strength to studies and allow results to be more easily validated. This will inevitably speed up the process of validating and qualifying biomarkers for use in clinical trials and in a clinical environment. While single marker studies using enzyme-linked immunosorbent assay (ELISA) and immunohistochemistry (IHC) are important, the novel, more sensitive discovery-type techniques, such as sequential window acquisition of all theoretical fragment ion spectra-mass spectrometry (SWATH-MS), would be well employed in hunting for significant biomarker panels.

An interesting observation from the results is the number of biomarkers investigated for each BIPEDS category. Most were investigated as burden of disease followed by diagnostic and prognostic markers in that order. In the “Hypothetical development of biomarkers” laid out by Bauer *et al*.,^3^ B, D and P categories are included in the stage before E. To reiterate the conclusion of the 2013 ESCEO review, no biomarker has yet been sufficiently qualified to aid in clinical trials- it would seem that there is still yet to be a marker sufficiently qualified for researchers to use for this purpose. This study has presented biomarkers that have shown statistically significant results in over 10 studies and biomarkers with AUCs of over 0.9. However, there is a huge variety of parameters being used to test these biomarkers in a variety of patients. A universal agreement on the most important parameters to be investigated for each of the BIPEDS categories would surely propel biomarker research forward considerably. After nearly 2 decades of molecular biomarker research it seems that the bottleneck is coming from a lack of coordination.

The main limitation of this study is that resources were collated from two databases only. It is possible that potentially applicable studies have not been identified from the search.

## Conclusion

In the past 5 years, research into biomarkers in osteoarthritis has continued to gain momentum. However, there is a lack of consensus on definition and methods of diagnosis and classification which is creating obstacles to research. A clear definition of eOA and a decision on important disease parameters will facilitate more appropriate research and allow the coalition of laboratories boasting different strengths. While many of the aims set out by the ESCEO have stipulated it clear research direction there are currently no single biomarkers that have been sufficiently validated for clinical use. Biomarker panels may provide a promising avenue for further evaluation.

## Supplemental Material

Supplemental_material – Supplemental material for Clinically Relevant Molecular Biomarkers for Use in Human Knee Osteoarthritis: A Systematic Review Click here for additional data file. Supplemental material, Supplemental_material for Clinically Relevant Molecular Biomarkers for Use in Human Knee Osteoarthritis: A Systematic Review by James G. Convill, Gwenllian F. Tawy, Anthony J. Freemont and Leela C. Biant in CARTILAGE
