# Supplementary material for: Clinically Relevant Molecular Biomarkers for Use in Human Knee Osteoarthritis: A Systematic Review
Source: Cartilage. 2020 Jul 17;13(1 Suppl):1511S–1531S. doi: 10.1177/1947603520941239 (PMC8808945; doi:10.1177/1947603520941239)
Supplement: Supplemental_material – Supplemental material for Clinically Relevant Molecular Biomarkers for Use in Human Knee Osteoarthritis: A Systematic Review [file Supplemental_material.pdf]

Appendix 1. Molecular Biomarkers where no statistical significance was found in a given publication.

Marker	Citation	BIPEDS classification
Basal bradykinin	85	B
C1M	15	P, D
	35	B
	31	D
C1, 2C	7	B, P, D
C2M	35	B
	86	B, P
C3M	26	B
	35	B
Cathepsin K	24	B
Clusterin	87	P
Coll2-1	22	B, D
Coll2-1NO2	7	B, P, D
COMP	7	B, P, D
	23	B
CPII	7	B, P, D
CRP	31	D
CRPM	28	B
CS846	33	B
CTX-I	68	B, D
CTX-II	86	B, P
hsCRP	35	B
	33	B
	61	B
IL-1	69	B
IL-6	69	B
IL-8	69	B
	62	B
IL-10	69	B
Lubricin	87	P
MMP-3	7	B, P, D
MMP-13	69	B
NTX-I	34	B, P, D
	41	B, D

Marker	Citation	BIPEDS classification
OC	[7]	B, P, D
	[33]	B
PDGF-BB	[68]	B, D
	[12]	B, D
PIIANP	[33]	B
PINP	[33]	B
	[34]	B, P, D
Resistin	[46]	P
Sclerostin	[54]	B, D
TNF-α	[62]	B
VEGF	[69]	B
	[76]	B, P

B- burden of disease, C1M- MMP-mediated degradation of type I collagen, C1M- MMP-mediated degradation of type 2 collagen, C2M- MMP-mediated degradation of type 2 collagen, C3M- MMP-mediated degradation of type 3 collagen, Coll2-1- α-helical region of type II collagen, Coll2-1 NO2- Nitrated epitope of the α-helical region of type II collagen, COMP- cartilage oligomeric matrix protein, CPII- marker for synthesis of type II procollagen, CRP- C-reactive protein, CRPM- matrix metalloproteinase-dependent degradation of C-reactive protein, CS846- Chondroitin sulfate epitope 846, CTXI- C-terminal crosslinked telopeptide type I collagen, CTXII- C-terminal crosslinked telopeptide type II collagen, D- diagnostic, hsCRP- high sensitivity C-reactive protein, IL- interleukin, MMP- matrix metalloproteinase, NTXI- crosslinked N-telopeptide of type I collagen, OC- osteocalcin, P- prognostic, PDGF-BB- Platelet-derived growth factor with two B subunits, PIIANP- Serum N-propeptide of collagen IIA, PINP- procollagen type I N propeptide, TNF-α- Tumour Necrosis Factor-α, VEGF- Vascular endothelial growth factor

Appendix 2. Study characteristics of studies included in review.

Study	NIH quality assessment grade	Source	Analysis method
[6]	Fair	S	ELISA
[7]	Good	S	ELISA
[8]	Fair	S	ELISA
[9]	Good	S	ELISA
[10]	Fair	Bo, C	IHC
[11]	Fair	SF, C	Luminex microbead-based suspension array
[12]	Fair	S, SF	Multiplex array
[13]	Fair	S	HPLC-MS
[14]	Fair	S	HPLC-MS
[15]	Fair	S	ELISA
[16]	Fair	S	LC-MS
[17]	Fair	S	LC-MS
[21]	Fair	S, SF	ELISA
[22]	Fair	S	ELISA
[23]	Poor	S, SF	ELISA
[24]	Good	S	ELISA
[25]	Fair	Bo, C	IHC
[26]	Fair	S	ELISA
[27]	Fair	S	ELISA
[28]	Poor	S	ELISA
[29]	Fair	SF	ELISA
[30]	Good	S	ELISA

31	Fair	S	ELISA
32	Good	U	ELISA
33	Fair	S	ELISA
34	Good	S	ELISA
35	Fair	S	ELISA
36	Good	S	ELISA
37	Good	U	ELISA
38	Good	U	ELISA
39	Fair	U	ELISA
40	Good	U	ELISA
41	Good	U	ELISA
42	Good	S	ELISA
43	Good	S	ELISA
44	Fair	S, SF	ELISA
45	Fair	S	ELISA
46	Good	S	ELISA
47	Poor	SF	SWATH-MS
48	Fair	S	ELISA
49	Fair	S, SF	ELISA
50	Fair	S, SF	ELISA
51	Fair	S, SF	ELISA
52	Fair	S, SF	ELISA
53	Poor	S, SF	ELISA
54	Fair	S, SF	ELISA
55	Fair	S	ELISA
56	Fair	S, SF	ELISA
57	Fair	S, SF	ELISA
58	Fair	S, SF	ELISA

59	Fair	C	IHC
60	Good	S	ELISA
61	Fair	S	ELISA
62	Fair	S	ELISA
63	Fair	SF	ELISA
64	Fair	S	ELISA
65	Good	S	ELISA
66	Fair	S, SF	ELISA
67	Fair	S, SF	ELISA
68	Fair	S	ELISA
69	Fair	SF	ELISA
70	Good	SF	ELISA
71	Fair	S, SF, C	ELISA, IHC
72	Fair	S, SF	ELISA
73	Fair	S, SF, C	LC-MRM
74	Poor	S, SF, U	ELISA
75	Fair	S, SF	ELISA
76	Good	S	ELISA
77	Fair	S, SF	ELISA
78	Fair	SF	ELISA
79	Fair	S, SF	ELISA
80	Poor	SF	Radioimmunoassay
81	Fair	S	ELISA
82	Fair	S, SF	ELISA
83	Fair	S, SF	ELISA
84	Fair	S, SF	ELISA
85	Fair	SF	ELISA
86	Good	U	ELISA

[87]	Good	SF	Multiplexed high throughput SRM

Bo- bone, C- cartilage, ELISA- enzyme-linked immunosorbent assay, HPLC-MS- High-performance liquid chromatography-mass spectrometry, IHC- immunohistochemistry, LC-MRM- liquid chromatography-multiple reaction monitoring, LC-MS- Liquid chromatography–mass spectrometry, S- serum, SF- synovial fluid, SRM- selected reaction monitoring, SWATH-MS- Sequential Window Acquisition of All Theoretical Mass Spectra, U- urine
